# HEAD START – an innovative training approach for life-long learning

**Published:** 2017-05-12

**Authors:** Demissie Tadesse, Isabella Montgomery, Girija Sankar

**Affiliations:** 1 MD, CBM Regional Eye Health Adviser, Addis Ababa, Ethiopia.; 2 International Coalition for Trachoma Control, UK.; 3 Assistant Director of Programs & Communications, International Trachoma Initiative Task Force for Global Health, Decatur, GA 30030 USA.

**An innovative global training approach called HEAD START is bridging the gap between theory and live surgery, building skills for new trainees as well as providing continuing professional development for experienced surgeons.**

**Find out more**

You can find out more about HEAD START in Future Learn's free *Eliminating trachoma* online course which aims to inform and support the personnel implementing and managing trachoma programmes at a district and community level. Visit **www.futurelearn.com/courses/** eliminating-trachoma to sign up. See all ICTC preferred practices relating to trichiasis on the coalition's website: **www.trachomacoalition.org/resources**

Repeated infection with *chlamydia trachomatis* leads to inturned eyelashes and painful trichiasis which has a profoundly negative impact on quality of life. It can be corrected by eyelid surgery but, if left untreated, can lead to irreversible low-vision and blindness.

Twenty years ago, Demissie Tadesse was a young doctor who had completed his ophthalmic training in Italy but did not have the opportunity to practise his surgical skills for trichiasis. He remembers the early days of working back in his home country of Ethiopia. Looking back, he can see how a practical step between the theory he learnt in the classroom and his first surgery on a human eyelid would have eased the transition from theory to live surgery.

A comprehensive training approach, HEAD START, aims to bridge this gap. The innovative method, which is a result of wide collaboration between many partners, includes a surgical mannequin on which surgeons can practise their surgical skills. It helps new trainee surgeons to build their skills and confidence before performing surgery on their first patients as well as providing useful refresher training and regular professional development for experienced surgeons. The comprehensive training approach covers all aspects of preferred practices on addressing trichiasis developed by the International Coalition for Trachoma Control (ICTC).

With the scale-up in programming to trachoma elimination by 2020, much attention has focused on increasing the quantity of surgeries performed and the training of surgeons. Since Demissie became involved with HEAD START three years ago, it has become a widely-adopted training approach. All major funding initiatives supporting national trachoma programmes now use this ICTC recommended and WHO endorsed approach for newly-trained and experienced surgeons. To maintain high-quality training standards, all training is undertaken by certified national master trainers directly, rather than through the previously widely used (but now discouraged) cascade approach. The WHO now recommends mannequin-based training for trichiasis surgery as part of new and refresher trichiasis surgery training programmes.

Now a certified global trainer, Demissie is playing a key role in disseminating the training and developing surgical skills with people of different backgrounds from all over the world, from Pakistan to Mozambique. In just a few years, he has seen how the approach is contributing to improving people's lives, “By following proper standards and certification, the HEAD START approach is putting global standards in the limelight. As a result, the impact will be very different because the focus of the training is on good quality outcomes, which will address the challenge of trichiasis recurrence that we have seen in the past.”

**Figure:**
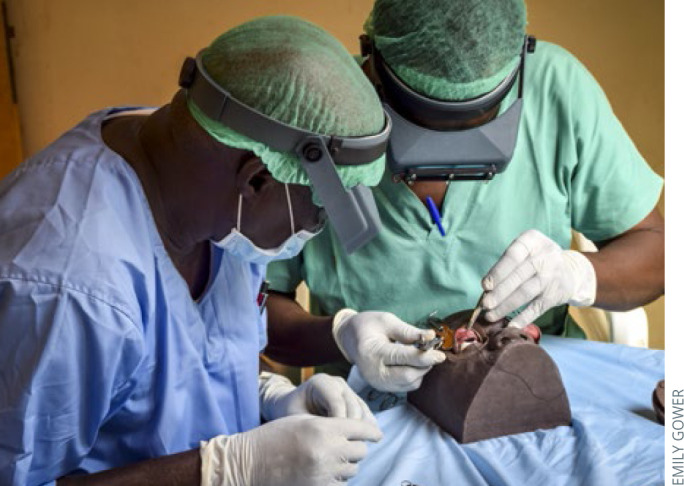
Surgeons undertaking training on the HEAD START mannequin.

Demissie has seen first-hand how practising on a mannequin allows surgeons to improve their work by allowing them to assess their technique. Both trainees and experienced surgeons have been able to improve their technique including angle of incision and suture placement, as all the steps can be revisited by removing the eyelid cartridge from the mannequin. Even experienced surgeons have been surprised by the improvements they were able to achieve. A recent trainee from Niger reflected, “The mannequin permits one to have surgical skills before applying them on humans; it's a good initiative for perfection.”

Emily Gower, epidemiologist and trachoma expert who has played a key role in developing the approach, remembers the expressions on people faces when the cartridges were taken out in the very first training session that took place in Tanzania, “You could see the wheels turning in the surgeons' heads and that they were thinking ‘I could do a better job by making these changes’.”

Going forward, a Seeing is Believing project in Ethiopia will support the maintenance of skills through remote monitoring of surgeons during the rainy seasons when fewer surgeries are performed. Over five months, the surgeons will undertake two surgeries a week on the mannequin and send the cartridges to a central office for review and monitoring. The innovative approach has also germinated thinking in other areas. USAID's Morbidity Management and Disability Prevention (MMDP) Project, inspired by HEAD START, is developing a similar mannequin training approach for lymphatic filariasis hydrocele surgical training.

The HEAD START approach is improving quality outcomes across the global trachoma elimination programme. Through its training of trainers and now also augmented by remote learning, it is truly providing a tool for continuing professional development and lifelong learning.

